# Implementation factors and their effect on e-Health service adoption in rural communities: a systematic literature review

**DOI:** 10.1186/1472-6963-13-19

**Published:** 2013-01-12

**Authors:** Eveline Hage, John P Roo, Marjolein AG van Offenbeek, Albert Boonstra

**Affiliations:** 1 Department of Innovation Management & Strategy, University of Groningen, PO box 800, 9700 AV, Groningen, Netherlands

**Keywords:** e-Health services, Rural, Implementation, Adoption, Context, Process, Content

## Abstract

**Background:**

An ageing population is seen as a threat to the quality of life and health in rural communities, and it is often assumed that e-Health services can address this issue. As successful e-Health implementation in organizations has proven difficult, this systematic literature review considers whether this is so for rural communities. This review identifies the critical implementation factors and, following the change model of Pettigrew and Whipp, classifies them in terms of “context”, “process”, and “content”. Through this lens, we analyze the empirical findings found in the literature to address the question: How do context, process, and content factors of e-Health implementation influence its adoption in rural communities?

**Methods:**

We conducted a systematic literature review. This review included papers that met six inclusion and exclusion criteria and had sufficient methodological quality. Findings were categorized in a classification matrix to identify promoting and restraining implementation factors and to explore whether any interactions between context, process, and content affect adoption.

**Results:**

Of the 5,896 abstracts initially identified, only 51 papers met all our criteria and were included in the review. We distinguished five different perspectives on rural e-Health implementation in these papers. Further, we list the context, process, and content implementation factors found to either promote or restrain rural e-Health adoption. Many implementation factors appear repeatedly, but there are also some contradictory results. Based on a further analysis of the papers’ findings, we argue that interaction effects between context, process, and content elements of change may explain these contradictory results. More specifically, three themes that appear crucial in e-Health implementation in rural communities surfaced: the dual effects of geographical isolation, the targeting of underprivileged groups, and the changes in ownership required for sustainable e-Health adoption.

**Conclusions:**

Rural e-Health implementation is an emerging, rapidly developing, field. Too often, e-Health adoption fails due to underestimating implementation factors and their interactions. We argue that rural e-Health implementation only leads to sustainable adoption (i.e. it “sticks”) when the implementation carefully considers and aligns the e-Health content (the “clicks”), the pre-existing structures in the context (the “bricks”), and the interventions in the implementation process (the “tricks”).

## Background

Recently, there has been increasing awareness of the societal consequences of aging. Around the world, fertility rates are dropping and life expectancy is increasing such that societies are faced with an ageing population [1]. Aging will not only increase the need for healthcare services, and consequently increase healthcare costs [2,3], it will have an impact on all spheres of human life: the economic, the social, as well as the political spheres [1,4].

Rural communities will be especially affected by aging as they are confronted with the out-migration of working-age adults from rural to urban areas and the in-migration of former urban dwellers, often at retirement age [1,5,6]. These demographic trends have raised concerns about the quality of life and health in rural communities [5,7]. e-Health services are seen as one solution to these concerns [8-16], with e-Health as diverse as web portals and domotica^a^[17,18], and possibly encompassing both core healthcare services and social innovation (see, for example, [19]). European policymakers are investing heavily in e-Health developments [20], but e-Health implementation is not always successful. While e-Health implementation within organizational settings is known to have adoption problems [21], few studies have addressed the peculiarities and particularities of e-Health in rural communities. Available studies report non- [22,23] or only partial [24,25] adoption. A systematic overview of e-Health implementation factors specific to rural communities, however, is lacking. The rural context deserves further study as rural communities may have greater need for e-Health services, not only because the aging process increases health care demand, but also because of local scarcity of alternative services and of health personnel. Moreover, implementation of e-Health services may be harder, due to e.g. lack of infrastructure. This makes rural e-Health implementation especially relevant and challenging at the same time. This the rationale for this study and leads to the following research aim.

### Study aim and overview

This systematic literature review aims to contribute to our understanding of the implementation factors that determine successful e-Health adoption in rural communities. Such an understanding could improve policies and strengthen programs directed at enhancing living conditions in rural communities and thereby the quality of life and health of their inhabitants. Following the change model of Pettigrew and Whipp [26], this review identifies and classifies implementation factors in terms of the “context”, “process”, and “content” of the health intervention studied. Moreover, we explore patterns in which these implementation factors merge to trace possible interactions between them.

Our research question is formulated as follows: *How do context, process, and content factors of e-Health implementation influence its adoption in rural communities?* To answer this question we need to know 1) what e-Health services are implemented in rural communities and for what purposes, and 2) which factors promote or restrain e-Health services adoption by the targeted group of residents?

In our review, the term ‘e-Health services’ refers to any interactive communication and information technology aimed at enhancing community quality of life and/or individual health outcomes. This wide-ranging definition was chosen for a number of reasons. First, since to the best of our knowledge no reviews have been conducted on rural e-Health implementation factors, providing a broad overview is a logical first step. At a later stage such an overview can act as a starting point for research targeted at specific types of e-Health implementation. Second, as the rural community is of special interest to this study, a general definition of e-Health allows us to pay extra attention to community-directed e-Health applications in addition to those directed at individual health. Thus, this definition allows including all potentially relevant e-Health initiatives in rural communities.

### Theoretical framework

In view of the dispersed nature of the e-Health implementation literature that originates from different scientific disciplines, we needed a flexible, but also solid framework to coherently organize the selected papers’ empirical findings. We draw on Pettigrew and Whipp’s classic model of strategic management of change [26], which has been widely applied in comparative case study research across many sectors and organizational contexts [27-29], as well as in studies on the implementation of innovations in healthcare [30,31]. This model generates insight by analyzing three interactive elements; “context”, “process”, and “content” that together shape any strategic change. A guiding assumption [32] is that not only the change content, i.e., the e-Health application, but also the change context and process have a role in explaining change outcomes, i.e., adoption outcomes. Much evidence supports this assumption [33,34], including the interactive nature of their explanatory roles [20,35,36]. In our search for implementation factors that either promote or restrain e-Health adoption in rural areas, this model allows to systematically assign each surfacing factor to one of these three robust, yet well-defined categories.

Change outcomes can be intended or unintended [37], and can affect the individual as well as the community as a whole. In order to apply Pettigrew and Whipp’s model to e-Health implementation in a rural community context, we translated their conceptual definitions into operational definitions closely fitting our research domain (see Table 1).

**Table 1:** Classification framework with conceptual definitions

**Interactive elements and their definitions**	**Factors within each element and their definitions**
**Rural context**	
Geographical area with low population density, limited resource bases, relative isolation, and cultural or ethnic homogeneity [38], and the accompanying political, economic, social, and technological developments.	*Socioeconomic variables -* The social and monetary environment in which the community is located.
	*Individual resources and capabilities -* Factors that influence the ability of rural residents to adopt e-Health.
	*A need for e-Health -* Situation where e-Health can substitute for services that have disappeared or supplement existing services in a way that rural residents perceive as useful.
	*Third party involvement* - Involvement of actors or stakeholders that do not belong to the targeted user group.
**Implementation Process**	
“Streams of activity across time” [26:39] undertaken with the aim of implementing e-Health.	*Implementation team -* Stakeholders that initiate or promote change (a single stakeholder or a coalition of stakeholders).
	*Implementation strategies -* Assumptions of how change needs to be executed, formulated with the aim to implement e-Health.
	*Bottom-up strategy -* Implementation strategy based on shared project ownership based on horizontal relationships between stakeholders.
	*Top-down strategy -* Implementation strategy based on centralized project ownership with vertical relationships between a single stakeholder and external actors.
	*Resource management -* Strategic allocation of scarce resources.
	*Conflict management -* Management of competing stakeholder interests as well as their ideas on the project.
	*People and organizational issues -* Problems among individuals and organizations that occur when implementing e-Health, such as with technical support.
**e-Health Content**	
Refers to any interactive communication and information technology aimed at enhancing the quality of life and/or health outcomes in the broadest sense [39].	*Project design -* The set of shared ideas about what the project is, including its aims, costs, and conditions for success.
	*e-Health design -* Technical and user features of the implemented e-Health.
	*Sustainability -* The enduring adoption of the e-Health content.
**Adoption Outcomes**	
The degree of adoption by the targeted group, leading to individual and community-level outcomes.	*Individual level adoption outcomes -* The effects that the implemented e-Health has on the individual’s health.
	*Community-level adoption outcomes -* The effects that the implemented e-Health has on the quality of life in the rural community.

The method section below explains the paper selection and search procedures applied as well as our classification and data analysis methods. In the results section, we then examine the research perspectives adopted in the selected papers, and analyze their empirical findings in terms of implementation factors that promote or restrain e-Health adoption. In the discussion section, we reflect on why the identified implementation factors might influence e-Health adoption in rural communities and how they may work together in doing so. This leads to potential areas for future research. Finally, we draw conclusions on what is known about the implementation and adoption of e-Health in rural communities.

## Methods

Aiming to increase understanding of the implementation factors that determine the success of e-Health adoption in rural communities, we conducted a systematic literature review. The review followed a thematic analysis approach, which is especially well equipped to handle both qualitative and quantitative data [40,41].

### Inclusion and exclusion criteria

Before starting our search, we defined six inclusion and exclusion criteria. Criteria concern study population (inclusion and exclusion criteria 1–2), type of e-Health intervention (3–4) and study type (5–6). The inclusion and exclusion criteria are: 1) the papers focused exclusively on rural communities, or explicitly made a distinction between urban and rural communities (for a definition of rural community see Table 1); 2) the papers focused on the rural community as a whole, not on a specific group or minor characteristic within the group (e.g. a specific disease); 3) e-Health was considered as an interactive mechanism (e-Health is further defined in Table 1); 4) there was a relationship between the three keyword categories such that category “c” influences category “a” in an environment defined by category “b” (keyword categories are laid out in the search strategy section); 5) they were empirical studies addressing implementation published in peer-reviewed scientific journals; 6) the papers were written in English.

### Search strategy

In order to ensure that this review encompassed all the relevant literature on the latest developments in e-Health adoption in rural communities the four authors formed an interdisciplinary research team. Together we outlined a specific search strategy that included five databases: “EBSCO1”, “EBSCO2” “Embase”, “MUSE”, and “Web of Science”. The “EBSCO” database was divided into two separate databases. EBSCO1 focuses on healthcare (“PsycINFO”, “CINAHL” and “MEDLINE”) while EBSCO2 provides a broader view (“Business Source Premier”, “Academic Search Premier”, “EconLit” and “SocINDEX”). The search was conducted using three categories of keywords: category a) “quality of life”, “social network”, “social cohesion”, “wellbeing”, “empower*”, “ownership”, “community participat*”; category b) “rural”, “deprived area”, “remote area”; and category c) “e-Health”, “e-care”, “tele*”, “ICT”, “information technology”, “communication technology”, “communication system”, “information system”. In each search, one keyword from each category was used, resulting in 168 search combinations.

A pilot research was conducted in the Web of Science database and detailed notes were kept of this process (including notes on exact search entry method and number of hits per search combination). Nevertheless, as the search engines of each database include slightly different search options, there was a short learning curve each time we started searching a new database. In order to acquire all relevant papers, we attempted to create the widest search possible.

In the database “EBSCO1” and “EBSCO2”, the search was carried out according to search strategy 1; keyword from “category a” (in the field of “left open”) and keyword from “category b” (in the field of “left open”) and keyword from “category c” (in the field of “left open”). Within this strategy only peer reviewed papers were allowed.

In the database “Embase”, the search was carried out according to search strategy 2; keyword from “category a” and keyword from “category b” and keyword form “category c”. No field limits were applied.

In the database “MUSE”, the search was carried out according to search strategy 3; keyword from “category a” (in the field of “all fields”) and keyword from “category b” (in the field of “all fields”) and keyword from “category c” (in the field of “all fields”).

In the database “Web of Science”, the search was carried out according to search strategy 4; keyword from “category a” (in the field of “Topic”) and keyword from “category b” (in the field of “Topic”) and keyword from “category c” (in the field of “Topic”). The last search was conducted on 31 May 2011. Identified studies were then divided among two researchers (EH and JPR) and separately analyzed. In order to reach a consensus and mutual understanding of the inclusion criteria, both researchers assessed and compared their interpretations. In this assessment, the researchers each independently selected three papers that they interpreted as highly relevant to the research question and therefore fitting the inclusion criteria, three papers that did not match the criteria, and three papers where the researcher was not sure whether to include or exclude the study. These papers were then evaluated by the other researcher. The research team discussed the differences in interpretations or doubts and this led to a further sharpening and refining of the inclusion criteria.

Alongside these inclusion and exclusion criteria, the papers were subjected to a quality assessment. Two methodological quality checklists, one focusing on qualitative research and the other on quantitative research, were applied. These quality checklists were based on previous checklists used in various research fields [42-45] reflecting the range of papers selected.

As with the selection process, the quality assessment was also conducted independently by the two researchers. During an initial quality assessment trial, the two researchers (EH and JPR) each evaluated four papers’ methodologies and then compared their conclusions. Since there were only minor deviations (in less than 10 percent of judgments), no corrective measures for assessing the methodological quality were taken.

As a final check on exhaustiveness, the reference lists of selected papers were scanned for any further relevant studies. In addition, we scanned reference lists of articles key to the papers under review.

### Data analysis

The resulting papers were each characterized in terms of the country or region of data collection, the research field, the research aim, and the type of research (qualitative/quantitative data, data collection method and number of cases/sample size). The papers were categorized according to the focus of the research question and data. A classification matrix was used to carefully map each paper’s focus, and we will show in the results section how the focus of the papers varied. Each paper’s empirical findings were categorized according to the classification matrix into factors belonging to “context”, “process”, and “content” elements, and related to the reported “adoption outcomes”. Finally, after analyzing the resulting promoting and restraining factors, propositions were formulated for further research.

## Results

### Included studies

**Figure 1:**
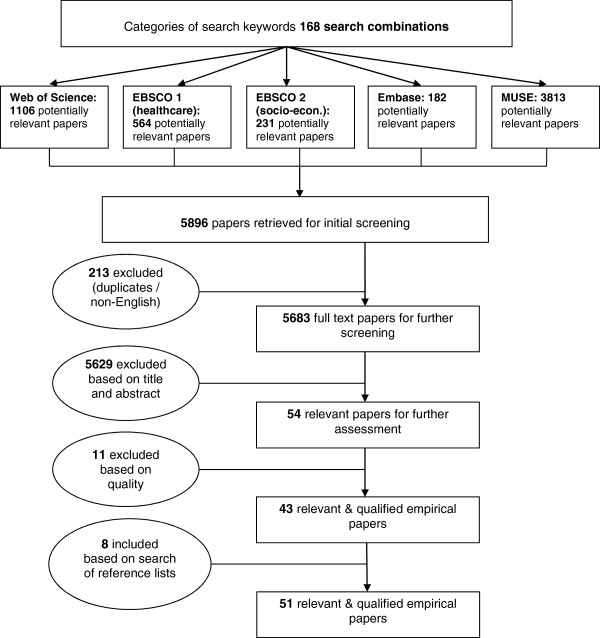
Flowchart of study selection process.

Through this search strategy, 5,896 papers were identified. After an initial screening we excluded 213 papers that were either duplicates or not written in English. Based on the title, abstract, and discussion, 5,629 papers of the 5,683 remaining papers were excluded because they did not meet all the inclusion and exclusion criteria 3–6 (see Inclusion and exclusion criteria subsection above). Of the 54 papers remaining, 11 were excluded because their quality was judged insufficient for our purposes (see Inclusion and exclusion criteria subsection). A search of the reference lists of these 43 included papers and of the reference lists of their key references yielded eight additional studies. Thus, our final sample amounted to 51 papers that met the inclusion criteria and sufficiently passed the quality assessment, see Figure 1. Additional file 1 presents the selected papers and their main results. Of the 51 relevant and qualified papers, 26 papers adopted a quantitative research approach, 14 used a qualitative research approach, and 11 papers used a mixed approach. Two papers [22,23] used the same data and analysis and were therefore jointly analyzed. Other papers that studied the same cases ( [46-48] and [49,50]) or used similar datasets ( [51-53], [54,55] and [56,57]) were analyzed separately as they were too dissimilar to combine.

Although some of the papers were more than ten years old, the majority were much more recent, with a sharp increase in relevant publications in 2010–11 (see Table 2). Rural e-Health implementation can thus be seen as an emerging and rapidly developing field.

**Table 2:** Number of papers published by period

**Year**	**Paper no.**
1995-99	47,74 *(n=2)*
2000-04	8,23,24,46,48,49,56,57,64,73,80,83 *(n=12)*
2005-09	11,12,13,22,25,50,51,53,54,58,59,62,65,68,69,71,77,78,79,81,82,85 *(n=22)*
2010-11	9,10,14,52,55,60,61,63,66,67,70,72,75,76,84 *(n=15)*

### *Sub-question 1:* What e-Health services are implemented in rural communities and for what purposes?

Thirty-five of the included papers reported on a specific e-Health implementation project. An example is a study on the effectiveness of a telehealth videoconferencing system [8]. The remaining 16 papers reported on the population level and studied e-Health adoption patterns and outcomes in general, yet within a specific population. For example, one study focused on general e-Health adoption outcomes based on healthcare practitioners’ perceptions of telehealth adoption in rural settings [58]. Both sets of papers focused on a variety of e-Health services, we discerned six categories:

1. *Internet and social media*[25,51-57,59-68] based services are developed for purposes ranging from economic development to empowerment and bridging the digital divide, as shown in the project level studies (n=7). The population level studies (n=11) usually focus on their adoption outcomes, especially social connectivity and access to information. These studies were conducted in both non-Western and Western oriented (North-America, Europe and Oceania) regions.

2. *Videoconferencing and telehealth*[8-10,13,14,22,23,58,69-74] are typically applied in services aiming to enhance the quality of life by improving both the accessibility and quality of those health services (project level n=10, population level n=4). We only found studies on videoconferencing and telehealth in Western oriented countries.

3. *Telecommunication applications (mobile phones*) [11,49,50,61,64,75-77]. Like internet and social media these are used to achieve a wide range of outcomes. Examples include saving health care costs by diagnosing a patient’s problems from mobile phone photographs, and enabling learning among rural women keeping goats by sending them voicemail messages (project level n=5); At the population level (n=3), telecommunication applications are mainly studied as a medium for reinforcing or changing social structures in view of long term health and wellbeing. A large share of the non-Western studies focused on projects implementing telecommunication applications.

4. *Community networks*[24,46-48,78-81] are analyzed for their ability to improve access to information, and particularly local information. In addition, community networks are believed to help bridge the digital divide (such as between rural and urban areas and between low and high income groups) and to empower rural communities (project level n=8). In addition to telecommunication, a large proportion of the non-Western studies focused on community networks.

5. *Web portals*[12,82,83] are usually believed to improve access to information, such as health-related and market information (project level n=3). The studies on web portals all originate from Western-oriented countries.

6. A *computer lab*[78,84,85] is usually implemented in a school setting and has an (e.g. health) educational purpose (project level n=3). Although small in number, these studies were conducted in Western and non-Western oriented regions.

Table 3 presents an overview of these six categories and links them to the e-Health outcomes targeted.

**Table 3:** Types and aims of e-Health

**Type of e-Health**	***Paper numbers***	**Aim related to**
Internet and social media	25,51,52,53,54,55,56,57,59,60,61,62,63,64,65,66,67,68	Social contact (51,52,53,60,61,63,66); Economic development (55,56,57,61,63,65,68); Access to information (52,53,62,64,67); Empowerment (55,57,59,61,65,67); Health (55,59,62,67); Bridging digital divide (25,63,54,67); Quality of life (general/other) (55,61,65); Education (55,61); Reducing costs/time (63).
Videoconferencing and telehealth	8,9,10,13,14,22,23,58,69,70,71,72,73,74	Health (8,9,10,13,14,22,23,58,69,70,71,72,73,74); Bridging the digital divide (13,14,58,69,70,71,72); Reducing costs/time (13,22,23,58,73,74); Education (8,10). Access to information (71); Social contact (10).
Telecommunication (mobiles)	11,49,50,61,64,75,76,77	Access to information (49,50,61,64); Education (61,75); Reduction cost/time (11,50); Health (11,61); Quality of life (general/other) (61,77). Bridging digital divide (50); Social contact (61); Economic development (61).
Community networks	24,46,47,48,78,79,80,81	Access to information (24,46,47,48,80,81); Bridging the digital divide (46,47,48,80); Empowerment (47,78,79,81); Reducing costs/time (24); Health (78); Economic development (78,79); Education (78); Social contact (78).
Web portal	12,82,83	Access to information (12,82,83); Health (12,82); Bridging the digital divide (83).
Computer laboratory	78,84,85	Education (78,84,85).

### Papers’ perspectives

**Figure 2:**
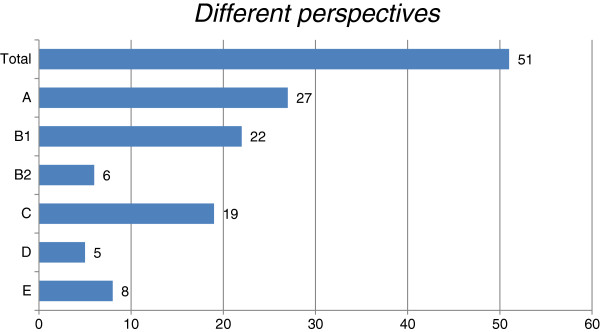
Number of papers per perspective.

The papers reflected different perspectives on how e-Health is adopted in rural settings and we were able to identify five categories (A, B, C, D, and E), which we outline below. While some papers took only one angle, others covered several perspectives. None of the papers involved all the categories. Figure 2 summarizes the number of papers per perspective (see also Additional file 1).

*A: Individual and community characteristics.* This perspective considers the individual and community level contextual factors (e.g. age, income, education level, social structures, local political climate) that influence e-Health adoption. Researchers adopting this perspective examine who is most likely to adopt e-Health. Linking this perspective to the elements of strategic change proposed by Pettigrew and Whipp [26] emphasizes ways in which contextual factors can influence e-Health adoption.

*B: e-Health shaping context.* This perspective focuses on the contextual effects of e-Health adoption and conceptualizes the relationship between e-Health content and context. Two sub-categories can be identified. Firstly, *B1* where effects are measured on the individual level and papers discuss how individuals have been affected by e-Health (e.g. in terms of access to information, wellbeing) and, secondly, *B2* where communities as a whole are the focus (e.g. wider social network).

*C: e-Health implementation.* Papers adopting this perspective address how e-Health services with specific characteristics are implemented in a particular context. The emphasis is on the *process* (i.e. activities) through which the e-Health content and the local context are adjusted to each other (e.g. through training or management strategies).

*D: Community shaping of e-Health.* This focus addresses how communities have participated during the implementation phase of an e-Health project. Here, the relationships between context and process, which were also present in the previous focus (C), are also applicable. The difference between perspectives C and D is that D emphasizes shared project ownership, whereas C emphasizes an implementation process that can have both centralized and shared project ownership.

*E: Individual appropriation of e-Health.* These papers focus on how individuals adopt e-Health. They address questions such as; how are e-Health services incorporated in an individual’s everyday life? The emphasis is on individuals finding appealing and innovative ways to use e-Health given the original functionalities. In terms of Pettigrew and Whipp’s strategic change elements, perspective E studies how the individual *context* affects the appropriation of the e-Health *content*.

Figure 2 shows that only a few of the 51 papers selected address perspectives B2, D, and E. This finding highlights the limited availability of data on how e-Health shapes the community, and how communities shape e-Health through their involvement in the implementation process and through individuals appropriating e-Health. Nevertheless, these perspectives are relevant as we will show in our discussion where we explore possible interaction effects between context, process, and content elements of change and develop propositions for future research.

### Sub-question 2: Which factors promote or restrain e-Health services adoption?

We have identified the factors that have been reported as either promoting or restraining e-Health adoption. Using Pettigrew and Whipp’s elements of change, these factors have been classified as context, process, or content factors. The factors are then categorized and linked to the paper’s reference number and perspectives.

### Context

Contextual factors promoting e-Health can be divided into three categories (see Table 4). The first category focuses on socioeconomic variables. Living in a geographically isolated area increases the need for “experiential information” [12], and therefore there is a positive relationship with e-Health adoption. Younger people and those with higher incomes are more likely to adopt e-Health. Further, family composition influences e-Health adoption (positively for married couples and families with children). Secondly, an individual’s resources and capabilities can be a promoting factor. When an individual has a network of non-local ties, e-Health can contribute by facilitating an easy way to communicate. Those with ICT skills, or those who are familiar with other technologies, are also more likely to adopt e-Health. Furthermore, if individuals are highly involved in their community they may identify e-Health as a public responsibility and start to maintain the e-Health service, and through this maintenance usage will increase. The third category emphasizes the need for e-Health. If alternative supply of information or services is low, e-Health can serve as a substitute. Needs that motivated people to adopt e-Health included: having greater anonymity, becoming self-reliant, helping others understand e-Health, and gaining access to information and services.

**Table 4:** Context factors promoting e-Health implementation

**Category**	***Factor***	**Paper no.**	**Paper perspective**
Socioeconomic variables	Geographical isolation	12,13	A,B1,E
	Demographics (low age, male, married, family composition includes children)	11,14,24,48,49,51,53,54, 60,62,63,69,79,83	A,B1,B2,C,E
	High occupation status, high income	47,48,49,53,54,62,63,64,79	A,B1,B2,E
Individual resources and capabilities	Having non-local ties	52,53	A,B2
	ICT experienced	14,47,48,54,72,82	A,B1,C
	Highly educated, high literacy	47,49,53,61,62,63,69,79	A,B1,B2,C,E
	Political and community involvement	46,47,48,52,53,85	A,B2,C,D
A need for e-Health	Lack of or barriers to services/information	8,12,13,24,52,59,78	A,B1,B2,C,E
	Fulfilling a specific need	13,25,49,54,76,59	A,B1,B2,C,D,E

Contextual factors restraining e-Health can be divided into three categories; socioeconomic variables, individual characteristics, and third parties (see Table 5). Some of the previously listed promoting factors are also present here as restraining factors in an antonymous form (i.e. the different influences of low and high incomes). Firstly, socioeconomic variables can function as a restriction when it comes to accessing e-Health (e.g. older population, low income/poverty, unemployment, geographical isolation). In addition, structural social inequalities (e.g. gender inequalities, caste system) are reflected in unequal e-Health access and therefore patterns in adoption. Secondly, individual resources and capabilities can be a restraining variable when they diminish the ability of an individual to adopt e-Health. Reasons can be a lack of ICT skills, illiteracy, having local rather than non-local ties (making it unnecessary to adopt e-Health to communicate), lack of mobility to explore non-local ties, and a negative self-perception. Thirdly, third parties can influence the ability of the individual to access e-Health. Relevant factors here include a negative relationship with teachers (such as women being afraid to go to training sessions because the teacher is too intimidating), unwilling and competing third parties (preventing the e-Health implementation achieving a solid form), and parties that facilitate alternative media (such that the demand for care or communication is met by other, non-e-Health, programs).

**Table 5:** Context factors restraining e-Health implementation

**Category**	***Factor***	**Paper no.**	**Paper perspective**
Socioeconomic variables	Demographics (high age, female, single, having no children)	11,14,24,48,49,51,53,54,60,62,63,69,83	A,B1,B2,C,E
	Unemployment, low occupation status, low income	24,46,47,49,54,57,60,83	A,B1,C,D,E
	Geographical isolated	9,24,62,63,64	A,B1,B2,C
	Gendered society, caste system	24,49,75	A,B1,C,D,E
Individual resources and capabilities	Lack of ICT skills	12,52,59,61,63,64,75,83	A,B1,B2,C,D,E
	Low educated, illiteracy	49,60,75,80,83	A,B1,B2,C,D,E
	Having local ties	51,52,53,66	A,B2,E
	Inadequate physical or mental condition	12,14,22,23,72	A,B1,C,E
Third party	Teacher/student hierarchy	75	B1,D,E
	Unwilling third party	24,52,60	A,B1,B2,C
	Available alternatives for receiving services/information	22,23,51	A,B1,C,E

### Process

Process factors promoting e-Health could be classified into four categories: implementation team; implementation practices; bottom-up strategy; and top-down strategy (see Table 6). Firstly, if the implementation team is regionally based this is considered to be a promoting factor because this will make it more likely that they understand the local issues and the villagers. Not surprisingly, when the project staff members are more capable, better skilled, and motivated this will have a positive effect on e-Health adoption. Secondly, the most valuable implementation practice is training the users so as to facilitate them in adopting e-Health. This also includes strategies to get users involved with e-Health. Moreover, best practices, quick wins, evaluation, and feedback are all factors that positively influence e-Health adoption. Thirdly, for the promoting factor of a “bottom-up strategy”, it is vital to work with the local community. Moreover, through cooperating with equal partners and politically active citizens, needs and roles can be aligned. Furthermore, it is important to have an unbiased mediator who focuses on crafting a sustainable e-Health implementation. Fourth, a top-down strategy can also be successful if it uses a planned diffusion strategy with an identified needs-based e-Health service. Through using the market place, economic factors are dominant in e-Health adoption. Further, collective learning can take place if one provides implementation leadership that communicates with diverse groups.

**Table 6:** Process factors promoting e-Health implementation

**Category**	***Factor***	**Paper no.**	**Paper perspective**
Implementation team	Regionally based implementation staff	22,23,80	B1,B2,C
	Capable, skilled, motivated implementation staff	22,23,70,77,84,85	A,B1,C,D
Implementation practices	Training	8,10,14,24,25,48,55,63,75,78,80,83,84, 85	A,B1,B2,C,D,E
	Implementation strategy to motivate people (both from within and without)	47,49,79,80	A,B1,B2,C,E
	Best practices	10,22,23,70,84,85	A,B1,C,D
	Quick wins	65,70	C,D
	Evaluation and feedback loops both bottom-up and top-down	22,23,25,84	B1,C,D
Bottom-up strategy	Work with existing local community networks	48,63	A,C
	Partnership: local residents as partners from an early stage add value and know their needs; objectives and roles should be transparent	65	C,D
	In publically financed projects, civic leaders need the support of politically active citizens	48	A
	Unbiased mediator role	25,65	C,D
	Use of pilot implementation projects	65,85	A,C,D
Top-down strategy	Planned diffusion strategy with a need-based product/service	58	B1
	When computer resources are left to the market place, economy factors will dominate	48	A
	Implementation leadership, creating collective learning through openness	80	B1,B2,C
	Top-down decision-making through local politicians	9	B1

The process factors found to restrain e-Health were assigned to three categories: insufficient resources; conflict potential; and people and organizational issues (see Table 7). The first category, insufficient resources, includes situations where projects lack the authority or financial means to improve vital parts of the implementation process. As a consequence, these projects lack the capability to be successfully completed. Secondly, the “conflict potential” category includes factors that reflect a lack of consensus and commitment among key stakeholders. Further, it includes the inadequate distribution of decision-making power (or ownership) among stakeholders. Thirdly, the “people and organizational issues” category describes problems among individuals and between organizations that can occur when implementing e-Health and includes factors related to technical support problems, logistical problems, and regulatory issues.

**Table 7:** Process factors restraining e-Health implementation

**Category**	***Factor***	**Paper no.**	**Paper perspective**
Insufficient resources	Projects that have no authority or financial means and lack the capability to improve vital parts of the implementation process	24,58,84	A,B1,C,D
Conflict potential	Lack of consensus, decision power, and commitment among key stakeholders	24,25,58	A,B1,C,D
People and organizational issues	Problems with technical support	24,78	A,B1,C
	Logistical problems	22,23,24,58,84	A,B1,C,D
	Regulatory issues	25	C,D

### Content

Three types of promoting factors in the content of e-Health can be discerned: project design; e-Health design; and sustainability (see Table 8). Firstly, the project design should contain a set of shared ideas about what the project is aiming for, the costs and the conditions for success. This will promote e-Health adoption when the design includes the following factors: the e-Health project is tailored to specific and agreed needs; the technology is publicly available and accessible; technological artifacts are similarly interpreted by stakeholders; and realistic and pragmatic goals are set for both development and adoption in line with the available funding. The second type of promoting factor in the e-Health content is an appropriate e-Health design, which essentially means that the technical design features have to fit the local context. Moreover, the technology needs to be reliable, flexible, mobile, ergonomic, user-friendly, and have a high image quality where applicable. The third group of factors concerns sustainable e-Health content adoption. If stakeholders are made contractual partners, they become part of the e-Health implementation process. Through such contracts, stakeholders commit to a long-term economic stake. Moreover, the collaboration among stakeholders will benefit when their roles are transparent, in terms of objectives, benefits, and outcomes, and this should be an essential part of the project design.

**Table 8:** Content factors promoting e-Health implementation

**Category**	***Factor***	**Paper no.**	**Paper perspective**
Project design	Tailored to specific and agreed upon needs	12,59,77,78,80,84	A,B1,B2,C,D,E
	Realistic and pragmatic goals	9,14, 64,70,71,72,77,78,83,84	A,B1,B2,C,D
	Funding and costs	70	C
	Availability	14,51,59,60,61,62,63,64,66,67,72,78,83	A,B1,B2,C,E
	Accessibility	11,14,24,51,61,64,79,80,82,84	A,B1,B2,C,D,E
	Distinctions between artifacts are commonly interpreted among relevant social groups, partners, and stakeholders	25	C,D
e-Health design	Designers considered local context in their design	22,23,24,25,48,75,79,83,84	A,B1,C,D,E
	Technological features	10,22,23,58	B1,C
Sustainability	Stakeholders should become contractual partners	65	C,D
	A community electronic network needs to sell itself	47	A

Content factors that restrain e-Health can be classified into two categories: project design; and e-Health design (see Table 9). The “project design” category explores those factors in the design of a project that restrict the adoption or usage of e-Health services. Here we find that low levels of availability and accessibility negatively affect e-Health adoption. The “e-Health design” category covers restraining factors related to design features of the e-Health service, including having e-Health services that do not meet a demand, funding problems, or an overly complex system that is difficult to use.

**Table 9:** Content factors restraining e-Health implementation

**Category**	***Factor***	**Paper no.**	**Paper perspective**
Project design	Funding and costs	9,14,25,64	A,B1,B2,C,D
	Low availability	75	B1,D,E
	Low accessibility	66	A,E
e-Health design	Not fulfilling a demand	9,52,60,61,63	A,B1,B2,C
	Poor user friendliness	52,59	A,B2,E

## Discussion

In the previous section we analyzed which factors promote or restrain e-Health service adoption within the context, process, and content elements of change.

To summarize, we have identified, in all the elements of change (context, process and content), key promoting and restraining factors related to e-Health adoption in rural communities. The identified factors in this review strongly relate to and cover Pettigrew and Whipp’s three original core elements of change [31]. However, the subcategories and especially their labeling sometimes had to be adapted as the original model was more often applied within organizational instead of community settings.

In some instances we found conflicting or limited results. Here, we first saw that geographical isolation features as both a promoting and as a restraining variable. Next, we saw that pre-existing socio-economic structures, such as structures of gender inequality and caste systems, complicate balanced diffusion of e-Health throughout the community. However, three studies [24,49,72,75] reported e-Health implementations that were able to diminish social inequalities. The question remains how these pre-existing socio-economic structures shape or get changed by ICT implementation. Finally, the studies show that local participation is essential in ensuring sustainability, but that it is not easy to manage. These three contradicting findings indicate that identifying promoting and restraining factors in itself does not provide solutions for these implementation issues. In this discussion we look at interaction effects to explain these results. While interaction effects have not been extensively studied in the papers reviewed, our theoretical framework [30] points towards explaining such seemingly contradictory results by taking a closer look at the interaction effects between the three change elements. We will discuss the contradictory findings below, but first address our review’s limitations.

### Limitations of this research

To our knowledge, no previous review has specifically analyzed the implementation factors that influence rural e-Health adoption. Notwithstanding the interesting results, this review has some limitations. Although we were careful in developing and executing our search strategy, the fact that e-Health implementation in rural communities is an emerging, and therefore broad and diffuse, field means that we cannot be sure that we have included all the relevant findings. The variety of terms used in this field may have limited our ability to achieve an exhaustive review. Moreover, for practical reasons this study excluded non-English papers. Papers that address the antecedents of successful e-Health implementation in general were not included either. Their relevance to the specific rural context may not always be evident and therefore, they fall outside the scope of this review.

A limitation of any literature review is that the authors of the studies selected will have had different objectives, and used different methods and means of interpretation in reaching their conclusions - conclusions which do not necessarily fully align with the interests of this article.

Although Pettigrew and Whipp’s model is parsimonious, flexible, much-cited, and widely applied, we might have chosen a different theoretical lens to bring together the empirical evidence on e-Health implementation in rural communities. However, their model not only served to systematically categorize the papers’ perspectives and the identified implementation factors, it also opened our eyes to the interaction effects between the change elements.

As the included papers vary greatly in research methods applied, it was impossible to conduct a meta-analysis. Rural e-Health is an emerging research area and by excluding qualitative papers from the review, the study would miss potentially relevant implementation factors. As it was, it was deemed important to include both quantitative and qualitative studies, instead of conducting a meta-analysis, therefore we conducted a thematic analysis [40,41] guided by the Pettigrew and Whipp-based classification matrix. We will now address the possible interaction effects suggested by our findings and their expected impact on e-Health implementation.

### Interaction between context and content: *the role of geographical isolation*

Although geographical isolation and the specific socioeconomic characteristics of rural communities (contextual factors) in general seem to restrain e-Health implementation, they can also create specific, contextualized needs that can be addressed with e-Health applications [8,12,13,22-25,49,51,52,59,76,78]. A few studies suggest that a needs-based e-Health content may compensate for the contextual factors that restrain e-Health implementation [8,82]. Needs-based e-Health applications seem to be able to overcome recognized socioeconomic ICT adoption barriers such as advanced age [11,14,48,49,51,53,54,60-63,69,83] and low incomes [24,46,47,53,54,62,63,69,83]. This relationship is especially well researched in Western oriented countries. Schmeida and McNeal [82], for example, found older people and individuals with low incomes to be more likely to use the internet in search of Medicare and Medicaid information than others. Moreover, in contrast to other studies [24,47,49,53,60-63,69,83], Schmeida and McNeil found no relationship between other personal characteristics, such as gender, race, ethnicity, and education, and online searches for Medicare or Medicaid information. Similar results are reported by Bynum et al. [8] who suggest that such findings “*may be explained by the limited access to quality health care knowledge among these groups*” [8]:220]. These findings suggest a strong interaction between content and context factors in determining e-Health adoption. As such, they constitute a warning against overgeneralizing the adoption effects of socioeconomic factors such as age, income, and education.

Furthermore, several studies argue that geographical isolation creates a need for e-Health because of a lack of alternative services or media in these areas [8,12,13]. Shepherd et al. [13] and Shaw et al. [12] address this need for particular subgroups. For example, Shaw et al. [12] show that individuals with poor well-being and little social support, i.e. those with a greater need for medical services, spend relatively more time on health information websites. Based on these findings, the authors claim that psychological help is of greater importance in rural communities because rural residents are more likely to feel geographically isolated from face-to-face support groups and therefore experience a greater need for e-Health solutions.

This creates a field of tension. On the one hand, rural users of e-Health applications may perceive e-Health as valuable and experience a concrete, valuable outcome from its use. On the other hand, the low network density, which defines geographical isolation, creates high barriers to sustainable e-Health implementation since it is difficult to make e-Health profitable in these circumstances. This is especially the case when targeting those with low incomes and of advanced age, groups which could most benefit from e-Health applications as is shown in [9,24,62-64].

Authors have formulated conditions under which public e-Health applications might be feasible, and suggested ways to provide incentives for private e-Health suppliers [25,48,55,65] (See also the later section on interaction between content and process). However, as these authors applied slightly different definitions of ‘rural’, further research is needed to test the proposed conditions and establish whether technology design can overcome the negative associations with socioeconomic trends. On this basis, we formulate the following proposition.

Proposition 1: Geographic isolation restrains e-Health implementation, yet provided that e-Health fulfills a specific need, geographical isolation promotes its subsequent adoption.

### Interaction between process and context: *how e-Health can add value for underprivileged groups*

As shown in the previous section, and indicated in the studies reviewed, there is an “*intriguing possibility that extant community structure […] may play an important mediating role in understanding the impact of internet access [or access to other ICT] on social relationships*” [46]:138]. Surprisingly, as shown in Figure 2, relatively little attention has been paid to the way these pre-existing socioeconomic community-level structures (i.e. context) affect the e-Health implementation process (i.e. process) and vice versa.

Although limited in number, some studies have considered the way pre-existing socioeconomic structures affect the process of e-Health implementation and adoption. Their general conclusion is that e-Health implementation usually reinforces rather than changes the pre-existing socioeconomic structures [49,52,53,64,66,76,79]. This reinforcement is in itself neither inherently positive nor negative, and may occur in quite subtle ways as illustrated by Gilbert et al. [66]. However, there are cases in which e-Health reinforces structures of socioeconomic inequality based on income, gender, age, and education. Forestier et al. [64] show, for instance, that income inequality nationally tends to increase with rising levels of telephone and internet penetration. Aminuzzaman [49] found that e-Health implementation could reinforce gender inequality, even with a specific e-Health application aimed at empowering women. Also Stern and Dillman [53] conclude, in reference to Norris [86,87], that e-Health *“is a vital tool for activating the active*” [53]:421]. None of the available findings suggest that the availability of e-Health in itself will change socioeconomic structures. The irony is that underprivileged groups, including relatively many older, rural adults, who might benefit most are, however, less likely to start using it.

However, under certain conditions, e-Health implementation does alter socioeconomic structures [24,49,75,79], enabling underprivileged groups to adopt e-Health. These projects were all implemented in regions characterized by forms of socio-economic inequality, e.g. gender-based, and located in Asian countries. Balasubraman [75] studied a project that did succeed in implementing an e-Health application for rural women in a context of gender inequality. This project was successful because, in addition to handing out e-Health applications, women were trained to use the application and supported in face-to-face discussion groups, and eventually enabled to participate in developing the e-Health content. These non-technological features of the project ensured that the e-Health application reached its target group. This example illustrates that social change will only be achieved through e-Health when the actors in the implementation process are aware of the existing socioeconomic structures and strongly driven to change these structures.

Proposition 2: e-Health implementation will reinforce the socioeconomic structures already in place in a rural community unless it includes interventions specifically aimed at changing these structures.

### Interaction between content and process: *sustainable e-Health implementation*

Remarkably, only a few studies, from different parts of the world, address sustainability [47,65,84]. Almost all studies ended after the initial implementation phase, leaving unanswered the question of whether and how e-Health content becomes sustainably embedded within a community.

Hosman and Fife’s study [65] is one of those few studies that explicitly deal with sustainability. They present several apparently essential conditions for sustainable rural e-Health implementation, most of which relate to leadership and the development of effective partnerships. While not focusing on sustainability, seven other papers also underline the importance of leadership and partnerships in the implementation process [24,25,65,70,75,80,84]. We will discuss their findings below in order to better understand how e-Health implementation may become sustainable.

Three of the studies emphasize the importance of equal, bottom-up partnerships [24,65,80]. Kanungo, in studying the implementation of local knowledge centers, for example, concludes that “*sustainability hinges on collaborative frameworks”*[80]:419]. Similarly, Cecchini and Raina [24], who studied a government-owned public computer network, ascribe its failure to governmental inability to establish an effective partnership with local communities and to allow these communities to acquire ownership of the e-Health project.

However, bottom-up partnerships alone may not be sufficient to ensure sustainable e-Health implementation as they also create complexity. Shin argued that calls for equal, bottom-up partnerships may be unrealistic as “*the possibility that one person’s success may be another person’s failure […] is rarely mentioned”*[25]:331]. In relation to this, Wit and Berner state “*the idea of mobilizing and organizing people collectively on the basis of horizontal [equal] ties and common interests does not appear to work well in most places, and, even more fatal, it appears to work less well the poorer and more dependent people are*” [88]:928]. This is the case because “*frequently, partnerships are asymmetrical, uneasy and often unsustainable, as they are based on personalized, vertical and informal relations that are frequently politicized, rather than on horizontal, collective relations rooted broadly in communities*” [88]:931]. This leads both Hosman [84] and Shin [25] to suggest that e-Health implementation requires a “*top-down-meets-bottom-up method*” [84]:38].

Following this line of thought, we suggest that bottom-up and top-down implementation strategies are in fact points on two continua: centralized versus shared ownership; and horizontal versus vertical relationships between the actors. Purely bottom-up or top-down implementation strategies are rare, if they exist at all, with most studies showing some in-between form. Further, as e-Health implementation is dynamic, implementation managers may decide to change their implementation strategy over the course of the implementation process. For example, a single stakeholder initiating an e-Health implementation may later want to partner other stakeholders to create broader support and ensure the project becomes embedded in the rural community. This entails a shift in implementation strategy from a horizontal, centralized ownership strategy to a horizontal or vertical shared ownership strategy. These results suggest that it is time to stop discussing the pros and cons of bottom-up versus top-down implementation strategies, and to start thinking about the strategic management of project ownership. By strategic, we mean that the implementation should involve well-timed shifts in project ownership with an eye on sustainable adoption. We therefore suggest the following proposition.

Proposition 3: In rural communities, sustainable e-Health adoption requires strategic changes in ownership over time.

## Conclusions

Rural e-Health [89] implementation is an emerging and rapidly developing field. This review paper shows that rural e-Health implementation could fail due to underestimating the implementation factors involved and the interactions between context, process, and content elements of change. For e-Health implementation to lead to sustainable adoption (i.e. it “sticks”), the e-Health content (the “clicks”) needs to align with the local contextual structures (the “bricks”) through strategic interventions in the implementation process (the “tricks”). While the type of technologies studied differed somewhat across the various regions included in the study, the general categories of implementation factors identified were almost the same. This led to the development of three propositions on the required alignment between these factors that will hopefully act as stimuli for further research.

### Endnotes

^a^ Domotics can be defined as “the set of elements that, when installed, interconnected and automatically controlled at home, release the user from the routine of intervening in everyday actions and, at the same time, provide optimized control of comfort, energy consumption, security and communications” [90];154].

## Competing interests

We declare that in the past five years we have not received reimbursements, fees, funding, or salary from an organization that may in any way gain or lose financially from the publication of this manuscript, either now or in the future. We do not hold any stocks or shares in an organization that may in any way gain or lose financially from the publication of this manuscript, either now or in the future. We do not hold, nor are we currently applying for any patents relating to the content of the manuscript. We have not received reimbursements, fees, funding, or salary from an organization that holds or has applied for patents relating to the content of the manuscript. Finally, we declare that we have no other competing interests (i.e. non-financial: political, personal, religious, ideological, academic, intellectual, commercial or any other) in relation to this manuscript.

## Authors’ contributions

EH was responsible for the research design, contributed substantially to the selection and analysis of included papers, and reworked an earlier version of the manuscript into the current article. JPR carried out a major share of the paper selection, contributed to their analysis, and wrote a preliminary draft of this article. MvO and AB made significant contributions to the framework and the interpretation of the results. They supervised EH and JPR throughout this study, and participated in writing the final version. All authors have read and approved the manuscript.

## Authors’ information

Eveline Hage is a PhD student in the Innovation Management & Strategy Department of the Economics and Business Faculty, University of Groningen. Focusing on the elderly, she studies conditions for ICT-enabled social change in rural communities.

John P. Roo holds master degrees in business administration change management and in human resource management. He is interested in strategic change and social interaction.

Marjolein A.G. van Offenbeek is an assistant professor in the Innovation Management & Strategy Department of the Economics and Business Faculty, University of Groningen. She studies the implementation and effects of new technologies, work structures, and professional roles in changing healthcare environments.

Albert Boonstra is a professor in the Organization and Innovation Department of the Economics and Business Faculty, University of Groningen. His research focuses on the implementation and use of complex information systems in healthcare environments and in particular on acceptance, resistance, stakeholder management, conflict, and use of power and politics.

## Pre-publication history

The pre-publication history for this paper can be accessed here:

http://www.biomedcentral.com/1472-6963/13/19/prepub

## Supplementary Material

Additional file 1 **Overview of papers included in the systematic literature review.** Additional file 1 provides an overview of the papers included in this systematic literature review, including information about year of publication, research perspective, research method, main dependent variable, type of e-Health service, and main results in terms of context, process, content (sub-) factors, and e-Health adoption outcomes. Click here for file
