# Supplementary material for: Implementation factors and their effect on e-Health service adoption in rural communities: a systematic literature review
Source: BMC Health Serv Res. 2013 Jan 12;13:19. doi: 10.1186/1472-6963-13-19 (PMC3575225; doi:10.1186/1472-6963-13-19)
Supplement: Additional file 1 — Overview of papers included in the systematic literature review. Additional file 1 provides an overview of the papers included in this systematic literature review, including information about year of publication, research perspective, research method, main dependent variable, type of e-Health service, and main results in terms of context, process, content (sub-) factors, and e-Health adoption outcomes. [file 1472-6963-13-19-S1.pdf]

Appendix A: Overview of papers included in the systematic literature review

Art .nr	Author	Year	Persp.	Context and study population	Research aim	E-Health intervention type & aim	Method	Main results
**52**	Aminuzzaman et al.	2003	A,B1,E	**Context:** Asia (Bangladesh)<br><br>**SP:** rural poor	Assessing the efficacy of the village phone in ameliorating the information poverty	Telecommunication (mobiles)<br><br>Providing modern telecommunication services to the poor people in Bangladesh	Multi-method: interviews, **N= Unknown**<br><br>survey **N=423 Response rate= Unknown**	**Context factors in e-Health adoption:**<br>Age (24-25yr)<br>Gender (literate men)<br>Education (literacy)<br>Traders and entrepreneurs (+)<br>Farmers (-)<br>**e-Health adoption outcomes:**<br>Ownership: Married housewives<br>Ownership: Age (35-55yr)<br>Time saving (+)<br>Social contacts migrant workers (+)<br>Uncertainty (-)<br>Anxiety (-)<br>Speed and quality decision making (+)<br>Health services (0)<br>Gender roles (0)<br>Empowerment higher in traditional regions

**62**	Balasubramanian et al.	2010	B1,D,E	**Context:** Asia (India)<br><br>**SP:** rural women	Understanding gender dimension in mobile phone learning tool	Telecommunication (mobiles)<br><br>Enhancing lifelong learning through mobile phones	Multi-method:<br><br>Interviews, focus group discussions, participant observations<br><br>**N= Unknown**<br><br>Survey<br>**N= 73**<br>**Response rate= 100%**	**Context factors in e-Health adoption:**<br>Low literacy levels (-)<br>Social hierarchy: teacher/student hierarchy (-)<br>**Process factors in e-Health adoption:**<br>Women participation (+)<br>Women ICT ownership (+)<br>Contextualization information (+)<br>Training (+)<br>F2F meetings (+)<br>Distribution through formal functions (reinforce ownership) (+)<br>Asking family members for help (+)<br>**Content factors in e-Health adoption:**<br>Costs calling/participating (-)<br>Mobile phone: learning & mobility (+)<br>**e-Health adoption outcomes:**<br>Using/bringing phone<br>Listening to voicemails<br>Applying information<br>Sharing voicemail information<br>Phone as symbol self-dependency

								Women maintain ownership (+)<br>Cognitive social capital (+)<br>Access market information (+)<br>Transport/opportunity costs (-)<br>Social network (+)<br>Calls to other participants (0)<br>In project participation (+)
**67**	Boase	2010	A	**Context:** North America (USA)<br><br>**SP:** American adults	Examining externality of personal networks in relation to low internet adoption in rural areas	(Broadband) internet and social media<br><br>No intervention	Survey<br>**N= 2200**<br>**Response rate= 35%**	**Context factors in e-Health adoption:**<br>Age (-)<br>Education (+)<br>Occupational status (+)<br>Occupational diversity (+)<br>Ties with internet access (+)<br>Single need e-mail (- high speed internet access)

**49**	Borgida et al.	2002	D	**Context:** North America (USA)<br><br>**SP:** rural communities	Investigating norms of cooperation and civic and political culture and (public) access to e-Health	Community networks<br><br>Increasing the community's access to and use of the national information infrastructure, sharing data and information with and between community members and partner organizations.	2 x Survey<br><br>(Survey 1)<br>**N= 2000**<br>**Response rate= 40%**<br><br>(Survey 2)<br>**N= 2791**<br>**Response rate= 64%**	**Context factors in e-Health adoption:**<br>High civic involvement (+)<br>Long history of civic organization (+)<br>ICT access perceived as market responsibility (-)<br>Low income (-)<br>Negative perceptions of ICT (-)<br>Community structures seems to mediate the impact of ICT access<br>**e-Health adoption outcomes:**<br>Digital divide (-)<br>Income no longer predicted e-Health adoption
**63**	Burrell	2010	B2,E	**Context:** Africa (Uganda)<br><br>**SP:** rural women	Understanding inequalities that emerge from informal sharing of mobile phones in Uganda	Telecommunication (mobiles)<br><br>Decreasing inequalities in access of mobile phones	Interviews<br>**N= 52**	**e-Health adoption outcome:**<br>Medium to preserve/ enhancing/challenge pre-existing social structures<br>Power struggle over phone<br>Gifting<br>In- and excluding sharing behavior (secrecy/hiding)<br>New, more individual communication lines lessen control husbands on communication wife<br>Systematic exclusion of women

**8**	Bynum et al.	2003	B1	**Context:** North America (USA)<br><br>**SP:** rural communities	Assessing differences in e-Health program satisfaction	Videoconferencing and telehealth<br><br>Providing more healthcare information and increase health	Survey<br>**N= 2567**<br>**Response rate= 69%**	**Context factors e-Health adoption (satisfaction):**<br>Rural population (+)<br>Minority populations (+)<br>Age (+)<br>Education (-)<br>Limited access health care knowledge (+)<br>**Process factors in e-Health adoption:**<br>Education methods (+)
**24**	Cecchini, Raina	2004	A,B1,C	**Context:** Asia (India)<br><br>**SP:** rural poor community	Assessing impact e-government project	Community networks<br><br>Increasing the efficiency of information distribution and access	Survey<br>**N= 105**<br>**Response rate= 100%**	**Context factors in e-Health adoption:**<br>Poverty (-)<br>geographical isolation (-)<br>Gender (women) (-)<br>Cast barriers (-)<br>**Process factors in e-Health adoption:**<br>Educated, willing operator (+)<br>Lack technical support (-)<br>Unclarity information (-)<br>**Content factors in e-Health adoption:**<br>Slow internet (-)<br>Uncertainty electricity (-)<br>Not need based (-)<br>No budget improve quality of service backend (-)<br>**e-Health adoption outcomes:**<br>Costs (-)<br>Harassment/corruption (-)

								Low use/awareness (-)<br>Insufficient revenues (-)
**53**	Chowdhury	2006	B1	**Context:** Asia (Bangladesh)<br><br>**SP:** rural households	Examining household market participation effects of telecommunication	Telecommunication (mobiles)<br><br>Increasing the access to information and reducing the search costs	Survey<br>**N= 284**<br>**Response rate= Unknown**	**e-Health adoption outcomes:**<br>Information seeking / transaction costs (-)<br>Informed business (+)<br>Informed social (+)<br>Market participation (+)
**9**	Ferrer-Roca et al.	2010	B1	**Context:** Europe (Spain)<br><br>**SP:** rural patients	Assessing quality of life effects of e-Health	Videoconferencing and telehealth<br><br>Decreasing overall waiting time, discomfort, financial or time costs.	Survey<br>**N= 800**<br>**Response rate= 81,9%**	**Process factors in e-Health adoption:**<br>Political top-down decision (+)<br>Resistance to use (-)<br>**Content factors in e-Health adoption:**<br>Simple ordinary videoconferencing (+)<br>**e-Health adoption outcomes:**<br>Health (0)<br>Quality of life (0)<br>Travel costs (-)<br>Time costs (-)<br>Workload (-)

**73**	Forestier et al.	2002	A,B2	**Context:** cross-country (Bangladesh, Botswana, Zimbabwe)<br><br>**SP:** the poor	Understanding how teledensity may affect economic inequality	(Broadband) internet and social media, telecommunication (mobiles)<br><br>Enhancing economic benefits	Survey (cross-country)<br>**N= Unknown**	**Context factors in e-Health adoption:**<br>Rural/low connection density (-)<br>Income (+)<br>Education (+)<br>**Process factors in e-Health adoption:**<br>Language barriers (-)<br>**Content factors in e-Health adoption:**<br>Costs (-)<br>**e-Health adoption outcomes:**<br>high initial teledensity and high teledensity growth increase inequality rich and poor and rural and urban<br>Quality of life(life expectancy, child mortality, literacy secondary school enrollment) (0)
**88**	Fortin	2003	B1	**Context:** North America (Canada)<br><br>**SP:** patients of a local hospital	Evaluating a telemedicine network	Videoconferencing and telehealth<br><br>Complementing the organization of health services locally	Semi-structured interviews<br>**N= 15**<br>Interviews<br>**N= 34**<br>Observation<br>Document analysis	**e-Health adoption outcomes:**<br>Speed obtaining health services/diagnosing (+)<br>Waiting time (-)<br>Time (-)<br>Costs (-)<br>Communication spouse/treating physician (+)<br>Knowledge professionals (+)

**61**	Gagnon et al.	2006	B1	**Context:** North America (Canada)<br><br>**SP:** physicians and managers in a remote area	Investigating perceptions of telehealth, physicians and managers	Videoconferencing and telehealth<br><br>Increasing access to, and quality of, health care services and to lower health system expenditures	Interviews **N=54**	**Perceptions factors e-Health adoption:**<br>User-friendly/Need based<br>Integration daily practice<br>Motivation patients<br>Participation professionals<br>Rules for reliability<br>Support organization<br>Organizational resources available<br>Integration workflow<br>Regional agreement stakeholders<br>**Perceptions e-Health adoption outcomes:**<br>Access (+)<br>Continuity (+)<br>Information/service availability (+)<br>Medical education (+)<br>Contact with Peers (+)<br>Feeling isolation (-)<br>Costs (-)<br>Fear replacement on site physician

**10**	Gibson et al.	2011	B1	**Context:** North America (Canada)<br><br>**SP:** rural first nation community members	Investigating perceptions of using videoconferencing for mental health services – telemental health	Videoconferencing and telehealth<br><br>Increasing access to healthcare services, client satisfaction, and decreased costs.	Interviews<br>**N= 59**<br><br>Quantitative analysis<br>**N= 53**<br>**Response rate= Unknown**	**Perceptions e-Health adoption outcomes:**<br>e-Health not appropriate (-)<br>Hard to establish trust (-)<br>Other priorities (-)<br>Concerns privacy (-)<br>Concerns help in case of crisis (-)<br>Increasing continuity (+)<br>Increasing access (+)<br>Reduction travel costs (+)<br>Foreseeing technological problems (-)
**75**	Gilbert et al.	2010	A,E	**Context:** North America (USA)<br><br>**SP:** rural and urban communities	Examining differences social media use rural and urban population	(Broadband) internet and social media<br><br>Connecting people, bonding and bridging	Content/Meta-analysis<br>**N= 4000**<br>**Response rate= 84,55%**	**Context factors in e-Health adoption (rural):**<br>Women (+)<br>Limited broadband access<br>Limited mobility<br>Necessity of trust<br>**e-Health adoption outcomes (rural):**<br>Moving f2f friendships online (+)<br>Meeting place (-)<br>Sign up later<br>Friends/comments (-)<br>Login (+)<br>Female most popular friend<br>Closeness friends (+)<br>Rural women's privacy measures (+)

**22/ 23**	Hebert et al.	2006 & 2004	B1,C	**Context:** North America (Canada)<br><br>**SP:** rural palliative care patients	Examining readiness to use influencing use, Effects on Symptom management and costs	Videoconferencing and telehealth<br><br>Increasing access to healthcare, reducing healthcare costs	Survey<br>**N= 44**<br>**Response rate= 13,75%**<br><br>Interviews<br>**N= 8**	**Context factors in e-Health adoption:**<br>Client motivation (+)<br>Nurses willingness to learn (+)<br>Alternative services (-)<br>critical mass (+)<br>Area size (-)<br>**Process factors in e-Health adoption:**<br>Management support (+)<br>Communication (+)<br>Experienced / regional team members<br>Difficult integration work pattern (-)<br>**Content factors in e-Health adoption:**<br>User-friendliness (+)<br>Technological failure (-)<br>Complex services (-)<br>**e-Health adoption outcomes:**<br>Non-adoption (0)<br>No effect (0)<br>Perception calls interruption nurse routine (-)

**60**	Hollifield et al.	2000	B1,C	**Context:** North America (USA)<br><br>**SP:** businesses and residents in two project and control communities	Examining whether rural telecommunications self-development projects were effective in encouraging local adoption of new telecommunication services	(Broadband) internet and social media<br><br>Empowering people by reducing social and cultural isolation and fostering economic development through implementing rural telecommunications self-development projects	Quasi-experiment<br><br>Interviews<br>**N= Unknown**<br><br>Survey<br>**N= 471**<br>**Response rate= 54%**	**Context factors in e-Health adoption:**<br>Self-employed (-)<br>Employed by local company (-)<br>**Process factors in e-Health adoption:**<br>Rural telecommunications self-development projects are not more effective then when telecommunication access is left to market forces<br>**e-Health adoption outcomes:**<br>Small community residents using the internet/e-mail at least once (+)<br>Small community businesses using the internet/e-mail at least once (-)<br>Using internet/e-mail at work (+)<br>Using company website (+)
**59**	Hollifield, Donnermeyer	2003	A	**Context:** North America (USA)<br><br>**SP:** individuals in four rural communities	Examining variables that influenced rural residents comparatively early adoption of information technologies	(Broadband) internet and social media<br><br>No intervention	Survey<br>**N= 471**<br>**Response rate= 54%**	**e-Health adoption outcomes:**<br>Employment by a company using specific information technology (+)<br>Employment by a company using specific information technology and low educated (+)

**79**	Hosman	2010	B1,C,D	**Context:** Africa (Uganda)<br><br>**SP:** rural community	Assessing conditions for introducing e-Health into primary and secondary schools	Computer lap<br><br>Increasing technological adoption.	Multi-method case study:<br><br>Interviews, **N= Unknown**<br><br>Content analysis	**Process factors in e-Health adoption:**<br>Setting realistic goals (+)<br>Taking local context into account (+)<br>Modest project size (+)<br>External (financial) support (+)<br>Training (both professionals and teachers) (+)<br>Project champion (+)<br>Bottom up and top down feedback loops (+)<br>Language barriers (-)<br>Limited capacity (-)<br>**Content factors in e-Health adoption:**<br>View e-Health as complementary instead of goal (+)<br>Sustainability<br>**e-Health adoption outcomes:**<br>Target group uses computers (+)<br>Enhancing learning (+)<br>Supporting local economy (+)

**74**	Hosman, Fife	2008	C,D	**Context:** Asia (Sri Lanka)<br><br>**SP:** rural communities	Exploring ways to design projects that are sustainable, enable long-term e-Health adoption an improve quality of life through public private partnerships	(Broadband) internet and social media<br><br>Encouraging local entrepreneurship and offering a variety of value adding training courses to address the socio economic desires of the local technology recipients.	Case study	**Process factors in e-Health adoption:**<br>Pilot project (in line with local realities, flexible)<br>Third party intermediary (managing stakeholders, solid contract, sustainability)<br>Bottom-up for need based approach (including understanding of societal norms, literacy levels, employment options, weather-related concerns, factional/religious/ethnic sensitivities, government openness or repression)<br>**Content factors in e-Health adoption:**<br>Services should be useful
**11**	Jayaraman et al.	2008	A,C	**Context:** Australia (New Zealand)<br><br>**SP:** rural patients and health professionals	Assessing usefulness and access to mobile phone cameras for diagnosing in primary care	Telecommunication (mobiles)<br><br>Assessing the clinical usefulness of mobile phone cameras	Survey<br>**N= 480**<br>**Response rate= Unknown**<br><br>Clinical quiz<br>**N= 30**	**Context factors in e-Health adoption:**<br>Maori-ethnicity (+)<br>Age (-)<br>Access (+)<br>Motivation (+)<br>**e-Health adoption outcomes:**<br>Increasing confidence in medical diagnose

**80**	Kanungo	2004	B1,B2, C	**Context:** Asia (India)<br><br>**SP:** rural community	Examining conditions for building emancipating e-Health	Community networks<br><br>Decreasing the information poverty	Interviews<br>**N= Unknown**	**Context factors in e-Health adoption:**<br>Clear government structures, local social structures may hinder even diffusion of e-Health adoption throughout community (due to i.e. literacy levels, time constraints)<br>**Process factors in e-Health adoption:**<br>Local, specific, community owned, partnerships<br>**Context factors in e-Health adoption:**<br>Need based<br>**e-Health adoption outcomes:**<br>Informing/knowledge sharing<br>Market access<br>Changed social order
**68**	Kivunike et al.	2011	A,C	**Context:** Africa (Uganda)<br><br>**SP:** rural community	Exploring perceptions of e-Health effecting quality of life in relation to e-Health adoption	(Broadband) internet and social media, telecommunication (mobiles)<br><br>Fostering economic growth, modernization and people centered or human development	Multi-method:<br><br>Interviews<br>**N= 22**<br><br>Survey<br>**N= 454**<br>**Response rate= 98%**	**Context factors in e-Health adoption:**<br>Perception of self (to old -)<br>Availability (+)<br>Education (+)<br>Perceived usefulness/need (+)<br>**e-Health adoption outcomes:**<br>Spam (-)<br>Time constrains (-)<br>Appropriation (Using e-

								Health for entertainment and communication rather than education)
**76**	Kumar, Best	2006	A,C	**Context:** Asia (India)<br><br>**SP: U**sers	Examining why kiosk use has not been able to diffuse among a wider section of the community population	Community networks<br><br>Supporting rural social, economic and political development	Survey<br>**N= 132**<br>**Response rate= Unknown**	**Context factors in e-Health adoption:**<br>Age (-)<br>Male (+)<br>Students (+)<br>Education (+)<br>Income (+)<br>Status community of origin (+)<br>**Process factors in e-Health adoption:**<br>Local champions (+)<br>**Content factors in e-Health adoption:**<br>Accessibility as centers are located close to economically backward neighborhoods (- existing socio-economic inequalities)<br>Localized content(+)

**57**	LaRose et al.	2007	A	**Context:** North America (USA)<br><br>**SP:** residents of four rural communities	Examining factors that cause broadband Internet service adoption in rural communities	(Broadband) internet and social media<br><br>Providing access to broadband	Survey<br>**N= Unknown**<br>**Response rate= between 20-47%**	**Context factors in e-Health adoption:**<br>Prior internet experience (+)<br>The expected outcomes of broadband usage (+)<br>Direct personal experience with broadband (+)<br>Self-efficacy (+)<br>Age (-)<br>Income (+)<br>Education (0)<br>Ethnicity (0)
**58**	LaRose et al.	2011	B1,B2, C	**Context:** North America (USA)<br><br>**SP:** rural residents	Examining adoption; awareness, perceptions and intentions to use; personal economic development; Community satisfaction	(Broadband) internet and social media<br><br>Providing access to broadband	Survey<br>**N= Unknown**<br>**Response rate= Unknown**	**Context factors in e-Health adoption:**<br>ICT experience/ownership (+)<br>**Process factors in e-Health adoption:**<br>Community education (+)<br>Grant (mixed results)<br>Offering to build public broadband attracts private investors<br>**e-Health adoption outcomes:**<br>Positive perceptions broadband benefit (+)<br>Pers.business initiatives (0)<br>Community satisfaction level (0)<br>Broadband awareness (0)<br>Intention to use (0)<br>Broadband perceptions (+)

**69**	Lear et al.	2009	A	**Context:** South America (Columbia)<br><br>**SP:** rural, agglomeration, and metropolitan area	Determining determinants of internet use/patterns of internet use for patients with cardiovascular disease	(Broadband) internet and social media<br><br>Developing ways to deal with increased demand on health care services.	Interviews<br>**N= 294**	**Context factors in e-Health adoption:**<br>Geographical isolation (-)<br>Computer ownership (+)<br>Gender (Men)<br>Age (-)<br>Income (+)<br>Education (+)<br>Marital status (Married)<br>Need for information<br>**e-Health adoption outcomes:**<br>understanding disease (+)<br>Informed decision making (+)
**89**	Madoc-Jones et al.	2007	B1,C	**Context:** Europe (UK)<br><br>**SP:** parents from local school	Examining perceptions of telephone support service	Telecommunication (mobiles)<br><br>Providing cheap additional educational services	Interviews<br>**N= 26**	**Process factors in e-Health adoption:**<br>Initial f2f meeting (+)<br>Need based, one-to-one communication (+)<br>Skilled operators (+)<br>Call schedules fitting client planning (+)<br>Desire non-time delineated support (-)<br>**e-Health adoption outcomes:**<br>Anxiety (-)<br>Depression/despair (-)
**85**	Mars	2010	A	**Context:** North America (USA)<br>**SP:** Indian reservation	Accessing email and internet for families of disabled Native Americans	(Broadband) internet and social media<br><br>No intervention	Survey<br>**N= 708**<br>**Response rate= 66%**	**e-Health adoption outcomes:**<br>Computer ownership levels<br>Frequency of use

**91**	McInerney	2005	A,C	**Context:** Europe (Ireland)<br><br>**SP:** local school	Describing process of incorporating technology into school curriculum	Computer lab<br><br>Enhancing the teaching, learning and curriculum experience	Multi-method:<br><br>Interviews<br>**N= 94**<br><br>Survey<br>**N= 360**<br>**Response rate= 45%**	**Process factors in e-Health adoption:**<br>Workshops (+)<br>High levels of voluntarism (+)<br>Pilot, expand later (+)<br>Champions (+)<br>Self-education/experimentation (+)<br>Training (+)<br>Student mentors (+)<br>Work pressure teachers (-)<br>**Content factors in e-Health adoption:**<br>No appropriate software for Children with special needs (-)
**70**	McQuaid et al.	2004	A,B1,C	**Context:** Europe (Scotland)<br><br>**SP:** jobseekers in rural areas	Assessing utility e-Health as information service unemployed people (rural and urban) based on their experiences and attitudes	Web portal<br><br>Improving the ability for the unemployed to search for jobs	Multi-method:<br><br>Interviews<br>**N=424**<br><br>Survey<br>**N= 300**<br>**Response rate= 22%**	**Context factors in e-Health adoption:**<br>Unemployed (-)<br>Age (-)<br>Negative attitudes (-)<br>Perceived technical skills (+)<br>Technical skills (+)<br>Education (+)<br>Income (+)<br>Accessibility (+)<br>**Process factors in e-Health adoption:**<br>Adjust to needs (+)<br>local dimension services (+)<br>Training (+)<br>Number of vacancies (+)<br>Contact agencies-indiv. (-)

**71**	Michailidis et al.	2011	A	**Context:** Europe (Greece)<br><br>**SP:** Rural heads of households	Classifying internet user types	(Broadband) internet and social media<br><br>Increasing flexibility and affordability	Survey<br>**N= 920**<br>**Response rate= 36,8%**	**Context factors in e-Health adoption:**<br>Previous ICT experience (+)<br>Skills (+)<br>Need/Necessity (+)<br>Fear (-)<br>Income (+)<br>Proximity urban area (+)<br>Household includes kids <18 (+)<br>Age (-)<br>Education (+)<br>Employees (>4 +)<br>**Process factors in e-Health adoption:**<br>work with local communities (+)<br>create need (+)<br>Vocational training (+)<br>New insights in technology<br>**Content factors in e-Health adoption:**<br>Availability (+)<br>Costs/time loss (-)<br>**e-Health adoption outcomes:**<br>Basic users<br>Socially interactive users<br>Farm oriented users

**83**	Pendleton et al.	2008	B1,C	**Context:** North America (USA)<br><br>**SP:** Teenagers	Examining satisfaction HIV/STD intervention f2f versus long distance	Videoconferencing and telehealth<br><br>Finding efficient manner to discuss crucial topics.	Survey<br>**N= 571**<br>**Response rate= Unknown**	**Process factors in e-Health adoption:**<br>Initial f2f meeting<br>**e-Health adoption outcomes:**<br>Leadership (-)<br>Warmth/connection (-)<br>Clarity (-)<br>Comfortability (-)<br>Exchange socio-emotional information (-)
**87**	Pigg and Crank	2005	B1,B2	**Context:** North America (USA)<br><br>**SP:** Five communities	Investigating impact of the development of ICT in rural communities that have based their local economic development strategy on the availability of ICT	(Broadband) internet and social media<br><br>Developing technological infrastructure	Case studies<br><br>Semi-structured interviews<br>**N= Unknown**<br>Content analysis	**e-Health adoption outcomes:**<br>Economic growth (0)
**90**	Reid et al.	1998	B1	**Context:** North America (Canada)<br><br>**SP:** Physicians and patients	Providing and evaluating telemedicine services for rural physicians and patients in Nova Scotia	Videoconferencing and telehealth<br><br>Providing patient care at limit cost	Longitudinal study<br><br>Surveys<br>**N= Unknown**<br>Focus groups<br>**N= Unknown**<br>Interviews with practitioners<br>**N= Unknown**<br>Content analysis	**e-Health adoption outcomes:**<br>Patient care (+/0)<br>Accessibility care (+)<br>Costs (-)

**50**	Riedel et al.	1998	A	**Context:** North America (USA)<br><br>**SP:** rural community	Assessing impact electronic community on social capital and electronic democracy	Community networks<br><br>Increasing access to information, enhancing their economic and social capabilities.	Multi-method:<br><br>Interviews<br>**N= Unknown**<br><br>Survey<br>**N= 2000**<br>**Response rate= 40%**	**Context factors in e-Health adoption:**<br>Household with kids <18<br>Aged <55<br>Computer usage (+)<br>Computer ownership (+)<br>Computer training -> computer comfort (+)<br>Support political active citizens (+)<br>**Process factors in e-Health adoption:**<br>Build on top of pre-existing social networks (+)
**84**	Rubinoff	2005	C,E	**Context:** Latin America<br><br>**SP:** Female community leaders participating in the project, Project staff	Showing that the analysis of increasingly complex cybernetworked development efforts can benefit from a the actor network approach	Community networks<br><br>Strengthing communication, women's empowerment through creation of online life histories of female leaders	Feminist research methodology<br><br>Participant interviews<br>**N= 21**<br>Project staff interviews<br>**N= Unknown**<br>Content analysis	**Process factors in e-Health adoption:**<br>Sometimes multiple actors were involved in the creation of life histories.<br>**Content factors in e-Health adoption:**<br>Project delivered privacy sensitive content<br>Project delivered subjective content
**66**	Schmeida, McNeal	2007	A	**Context:** North America (USA)<br><br>**SP:** Users of a certain website	Examining disparities accessing health information through internet	Web portal<br><br>Providing specified healthcare information	Survey<br>**N= 2928**<br>**Response rate= 72,2%**	**e-Health adoption:**<br>Home internet access (+)<br>Age (+)<br>Income (-)<br>Duration internet access (+)

**12**	Shaw et al.	2008	A,E	**Context:** North America (USA)<br><br>**SP:** rural breast cancer patients	Investigating psycho-social factors influencing use and type of use of e-Health	Web portal<br><br>Providing specified healthcare information	Survey<br>**N= 144**<br>**Response rate= 92%**	**Context factors in e-Health adoption:**<br>Need for information (+)<br>Negative emotions (+)<br>Perceived barrier to information (+)<br>Worse condition (+)<br>Perceived social support (-)<br>Emotional wellbeing (-)<br>Sense of competence/self-efficacy (+)<br>Coping mechanism (+)<br>Reduced functional wellbeing (-)<br>**e-Health adoption outcomes:**<br>Preference experiential data
**13**	Shepherd et al.	2006	B1	**Context:** Australia<br><br>**SP:** rural cancer patients	Assessing effect of telepsychology on anxiety, depression and quality of life	Videoconferencing and telehealth<br><br>Providing mental supporting services, increasing healthcare quality, decreasing costs	Survey<br>**N= 34**<br>**Response rate= 74%**	**Context factors in e-Health adoption:**<br>Lack of specialist care/treatment options (+)<br>Geographical isolation (+)<br>Desire to be anonymous (+)<br>Effect of Context factors in e-Health adoption:<br>Travel costs (-)<br>Anxiety (-)<br>Depression (-)<br>Quality of life (Functional and emotional wellbeing) (+)<br>Physical wellbeing (0/+)<br>Social wellbeing (0/+)

**25**	Shin	2008	C,D	**Context:** North America (USA)<br><br>**SP:** rural community and its variety of stakeholders	Investigating development, discourse, design of community network and social interactions	(Broadband) internet and social media<br><br>Preparing the community for future economic and social opportunities	Interviews<br>**N= 66**	**Process factors in e-Health adoption:**<br>Community consensus/Alignment of ideas (+)<br>Communication project plan to community (-)<br>Mediating role (+)<br>Regulatory obstacles (-)<br>Not involving all stakeholders in decision process(-)<br>Emphasis of project's technological above community needs<br>Organizational politics (-)<br>**Content factors in e-Health adoption:**<br>Need-based (+)<br>Lack of technical and financial resources (-)

**14**	Simms et al.	2011	A,C	**Context:** North America (Canada)<br><br>**SP:** clinicians	Identifying factors associated with practitioners telemental health use, perceptions and barriers to use	Videoconferencing and telehealth<br><br>Increasing healthcare access and reducing healthcare costs	Multi-method:<br><br>Interviews<br>**N= 25**<br><br>Survey<br>**N= 160**<br>**Response rate= Unknown**	**Context factors in e-Health adoption:**<br>Illness symptoms (emotional unstable, impulsive, poor coping skills, cognitive impairments, psychotics, paranoia, particular mental illness)<br>Impaired physical abilities (-)<br>Trust issues (-)<br>Infrastructure (+)<br>Accessibility (+)<br>ICT experience (+)<br>Age (-)<br>Perceived user-friendliness<br>**Process factors in e-Health adoption:**<br>Facilitating emergency help<br>Training<br>Building trust<br>**Content factors in e-Health adoption:**<br>Lack of funding

**81**	Singh et al.	2010	C	**Context:** North America (USA)<br><br>**SP:** principal actors associated with the telehealth initiative	Assessing possibilities for sustainable adoption of e-Health by rural public health institutions	Videoconferencing and telehealth<br><br>Increasing access to and improvement of healthcare services in rural areas.	Interviews<br>**N= 25**	**Process factors in e-Health adoption:**<br>f2f still possible (+)<br>Leadership skills (+)<br>Support funding agencies (+)<br>Independent telehealth network<br>Collaboration health institute, rural community and external partners<br>**Context factors in e-Health adoption:**<br>Outreach clinics (+)<br>Costs (-)<br>**e-Health adoption outcomes:**<br>Access to new services<br>Decrease nurse turnover
**72**	Sørensen	2008	A	**Context:** Europe (Denmark)<br><br>**SP:** heads of rural households	Examining attitudes towards e-Health	Videoconferencing and telehealth<br><br>Increasing access to healthcare services in rural areas.	Survey<br>**N= 1000**<br>**Response rate= 64,3%**	**Context factors in e-Health adoption:**<br>Age (18-29, +)<br>Education (+)<br>**e-Health adoption outcomes:**<br>Reluctance applications patient-doctor procedures

**54**	Stern	2008	A,E	**Context:** North America (USA)<br><br>**SP:** rural community members	Defining modes of communication between rural individuals and three closest friends, looking at social tie locality, frequency of communication, internet usage	(Broadband) internet and social media<br><br>Increasing the degree of communication between people	Survey<br>**N= 1315**<br>**Response rate= 69%**	**Context factors in e-Health adoption:**<br>Alternative media (-)<br>low access (-)<br>local friends/f2f com (-)<br>Age (-)<br>Competence<br>Effect Context factors in e-Health adoption:<br>The more people use the internet, the more likely they are to use email in contact with close friends (both local and non-local), only if persons are proficient with internet.

**55**	Stern, Adams	2010	A,B2	**Context:** North America (USA)<br><br>**SP:** rural community members	Assessing how people use internet to maintain local social networks/learn about local activities	(Broadband) internet and social media<br><br>Increasing the degree of communication between people in which knowledge of community members about local activities in enhanced	Survey<br>**N= 1315**<br>**Response rate= 69%**	**Context factors in e-Health adoption:**<br>Non-local ties (+)<br>Local leadership and participation (+)<br>Local ties (-)<br>Religious groups (-)<br>lack of skills (-)<br>Alternative media (-)<br>Negative perceptions of ICT among non-users (-)<br>**Content factors in e-Health adoption:**<br>Non user friendly (-)<br>Fulfilling a specific need (+)<br>**e-Health adoption outcomes:**<br>Internet for bonding (mostly) and bridging<br>Activating the active<br>Email rather than website fosters community interaction

**56**	Stern, Dillman	2006	A,B2	**Context:** North America (USA)<br><br>**SP:** rural community members	Investigating involvement in local community activities, community leadership, local affective relationships affecting internet use	(Broadband) internet and social media<br><br>Increasing the degree of communication between people	Survey<br>**N= 1315**<br>**Response rate= 69%**	**Context factors in e-Health adoption:**<br>Age (-)<br>Education (+)<br>Marital status (married)<br>Income (+)<br>**e-Health adoption outcomes:**<br>Participation community events (0)<br>Participation community groups (+)<br>Participation community change (+)<br>Being a leader (+)<br>Non-local ties (+)<br>Internet used to organize those who are already civically engaged/with external network contacts
**51**	Sullivan et al.	2002	A	**Context:** North America (USA)<br><br>**SP:** rural community	Identifying individual and community characteristics influencing e-Health content	Community networks<br><br>Providing a foundation to enhance the community's economic, political and social resources	Survey<br>**N= 805**<br>**Response rate= 40,25%**	**Context factors in e-Health adoption:**<br>Household with kids <18<br>Aged <55<br>Computer usage (+)<br>Computer ownership (+)<br>Computer training -> computer comfort (+)<br>**Process factors in e-Health adoption:**<br>Support political active citizens (+)<br>Build on top of pre-existing social networks (+)

**64**	Wathen, Harris	2007	A,E	**Context:** North America (Canada)<br><br>**SP:** rural women	Exploring women's health seeking role in rural setting with e-Health media	(Broadband) internet and social media<br><br>Maintaining quality healthcare while services are scaled back or cut	Interviews<br>**N= 40**	**Context factors in e-Health adoption:**<br>Availability (+)<br>Lack of alternative information sources (+)<br>Perceived information quality (+)<br>Search skills (+)<br>Desire to be self-reliant (+)<br>Not want to bother others (+)<br>Confidence in information source (+)<br>**Content factors in e-Health adoption:**<br>Fulfills need (+)<br>Complexity of information (-)
**65**	Zhao	2008	A,B1	**Context:** Asia (China)<br><br>**SP:** rural farmers	Assessing ways in which internet use effects rural development (economic conditions and education) and social structures effects internet diffusion, adoption and use	Community networks, computer lab<br><br>Increasing economic and education conditions	Interviews<br>**N= 29**	**Process factors in e-Health adoption:**<br>Training (+)<br>Technical support (+)<br>**Content factors in e-Health adoption:**<br>Costs (-)<br>**e-Health adoption outcomes:**<br>Per capita income (+)<br>Aligning output-market (+)<br>Business opportunities and household earning (+)<br>Teaching qualities (+)<br>Learning capability (+)<br>Adult ICT readiness (+)<br>Digital divide rural/urban (-)

**86**	Zilliacus et al.	2010	A,B1	**Context:** Australia<br><br>**SP:** women breast cancer patients	Examining satisfaction, perceptions and quality of e-Health	Videoconferencing and telehealth<br><br>Increasing access to healthcare services	Interviews<br>**N= 12**	**Context factors in e-Health adoption:**<br>Complex cases (-)<br>**e-Health adoption outcomes:**<br>Telepresence increased ICT comfortability<br>Emotional support<br>Reduction travel/time costs

Appendix A provides an overview of the papers included in this systematic literature review. It includes information about year of publication, research perspective, research method, main dependent variable, type of e-Health service, and main results in terms of context, process, content (sub-)factors and e-Health adoption outcomes.
